# Transcatheter Uterine Artery Embolisation in Treating Secondary Haemorrhage Post Hysterectomy: A Life-Saving Approach

**DOI:** 10.7759/cureus.30249

**Published:** 2022-10-13

**Authors:** Nidhi A Patel, Neema Acharya, Kiran Borkar

**Affiliations:** 1 Department of Obstetrics and Gynaecology, Jawaharlal Nehru Medical College, Datta Meghe Institute of Medical Sciences, Wardha, IND

**Keywords:** hemorrhagic shock, transcatheter angiographic embolization, uterine artery embolization (uae), haemorrhage, hysterectomy

## Abstract

Hysterectomy, which is a surgical procedure to remove all or a part of the uterus, is one of the most commonly done procedures by a gynaecologist. However, it may be associated with a number of complications. Haemorrhage following hysterectomy is a life-threatening complication. One of the most common complications is haemorrhage, whether primary or secondary. The various options to treat secondary haemorrhage post hysterectomy are uterine artery embolization (UAE) or surgical re-exploration. Here, we present a case of secondary haemorrhage post hysterectomy treated with uterine artery embolization and describe the numerous advantages of UAE as a novel approach to stop bleeding post hysterectomy over the traditional surgical re-exploration method.

## Introduction

Hysterectomy, which is a surgical procedure to remove all or a part of the uterus, is one of the most commonly done procedures by a gynaecologist. It can be done via various approaches, including abdominal, vaginal or the recently advocated technique of assistance with laparoscopy. Hysterectomy can be total hysterectomy, subtotal hysterectomy, total hysterectomy and bilateral salpingo-oophorectomy (BSO), subtotal hysterectomy and BSO, or Radical/Wertheim’s hysterectomy. [1].

There are numerous Indications for performing abdominal hysterectomy like abnormal uterine bleeding, adenomyosis, endometriosis, neoplasia, etc. [2]. However, hysterectomy, like any other surgical procedure, is not without complications and can lead to sepsis, haemorrhage, venous thromboembolism, injury to the gastrointestinal tract or genito-urinary system, nerve injury and vaginal cuff dehiscence [3].

Haemorrhage is defined as a copious or heavy discharge of blood from a damaged blood vessel [4]. Postoperative haemorrhage can be primary, reactive, or secondary bleeding. Primary haemorrhage is defined as the one occurring at the time of surgery intraoperatively during dissection due to injury to a blood vessel or immediate postoperatively, while reactionary haemorrhage is the one occurring within 6-24 hours of surgery due to slippage of ligature, dislodgement of clot or cessation of reflex vasospasm. Secondary haemorrhage refers to bleeding occurring within 7-14 days after the operation due to sloughing out of blood vessels due to various reasons like infection, pressure necrosis or malignancy [5].

## Case presentation

Here we present a case of a 47-year-old married female who came to the emergency casualty department of our hospital with an obstetric score of P2L2A1. She was a housewife by occupation and came with complain of bleeding per vaginum (p/v) for 6 hours. Vitally, her blood pressure was 100/60 mmHg and was tachycardiac at 108 bpm and her SpO2 was 95% on room air. Examination of the cardiovascular and respiratory systems did not reveal any abnormalities. On per abdomen examination, the abdomen was soft but there was a swelling palpable in the right iliac fossa and hypogastrium of about 5 x 4 cm size. A lower transverse scar of total abdominal hysterectomy was visible over the anterior abdomen; however, there was no tenderness, guarding or rigidity and there was no evidence of any organomegaly. On per speculum examination, acute spontaneous oozing of blood through the vault of the vagina was present with plenty of blood clots in the vaginal canal. Per vaginal examination could not be performed due to excessive bleeding. The patient was stabilized with oxygen supplementation and IV crystalloid fluid support. Blood products including one packed red cell and one fresh frozen plasma were transfused to the patient. Vaginal packing was done in the casualty department to control bleeding as a temporary measure. On stabilization of the patient, her history was confirmed.

The patient had undergone total abdominal hysterectomy + bilateral salpingo-oophorectomy in view of symptomatic uterine fibroids at a private hospital. On postoperative day 2 of the surgery, she experienced massive bleeding per vaginum for which exploratory laparotomy was done and bleeding controlled. The patient was then discharged on day 4 of total abdominal hysterectomy + bilateral salpingo-oophorectomy and day 2 of exploratory laparotomy. 15 days after the discharge from the hospital, the patient again suffered bleeding p/v and was referred to a tertiary care centre in view of possible blood coagulation disorder. It revealed anaemia with leukocytosis and a normal coagulation profile with normal liver and renal function tests (Table 1).

**Table 1:** Patient's laboratory investigations on admission CBC: complete blood count; Hb: haemogolbin; MCV: mean corpuscular volume; TLC- total leukocyte count; Plt: platelets; PT: prothrombin time; INR: international normalised ratio; APTT: activated prothrombin time; KFT: kidney function test; LFT: liver function test

Investigations	Values
CBC	
Hb	5.4 gm/dl
MCV	75 fl
TLC	16,600/dl
Plt	3.31 lakhs/dl
Coagulation profile	
PT	13.7
INR	1.09
APTT	30.7
KFT	
Urea	17 mg/dl
Creatinine	0.5 mg/dl
Sodium	137 mmol/dl
Potassium	4.1 mmol/dl
LFT	
Total protein	5.9 gm/dl
Albumin	2.7 gm/dl
Globulin	3.2 gm/dl
Aspartate aminotransferase	28 units/dl
Alanine aminotransferase	16 units/dl
Alkaline phosphatase	62 IU/l
Total bilirubin	0.5 mg/dl

**Figure 1:**
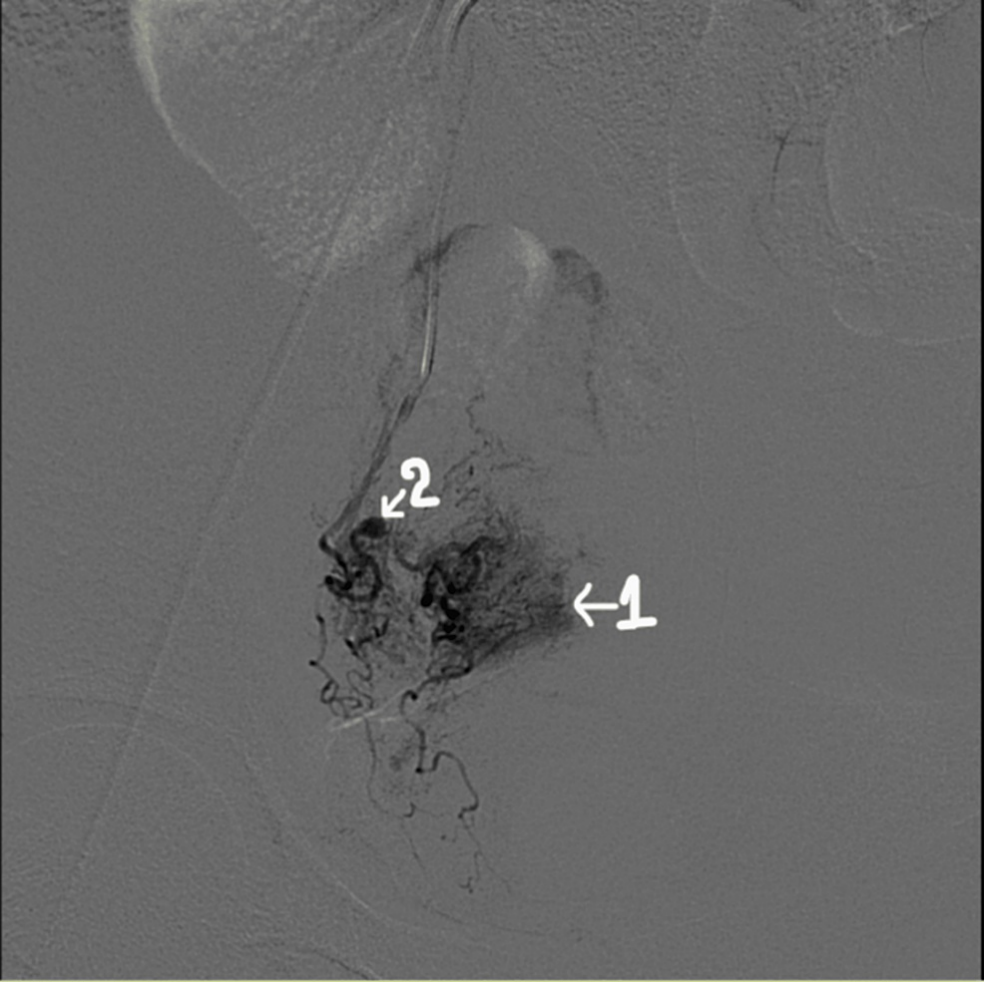
Pre-embolization spillage of dye (arrow 1) through right uterine artery branches and a small aneurysm in one of its branches (arrow 2)

**Figure 2:**
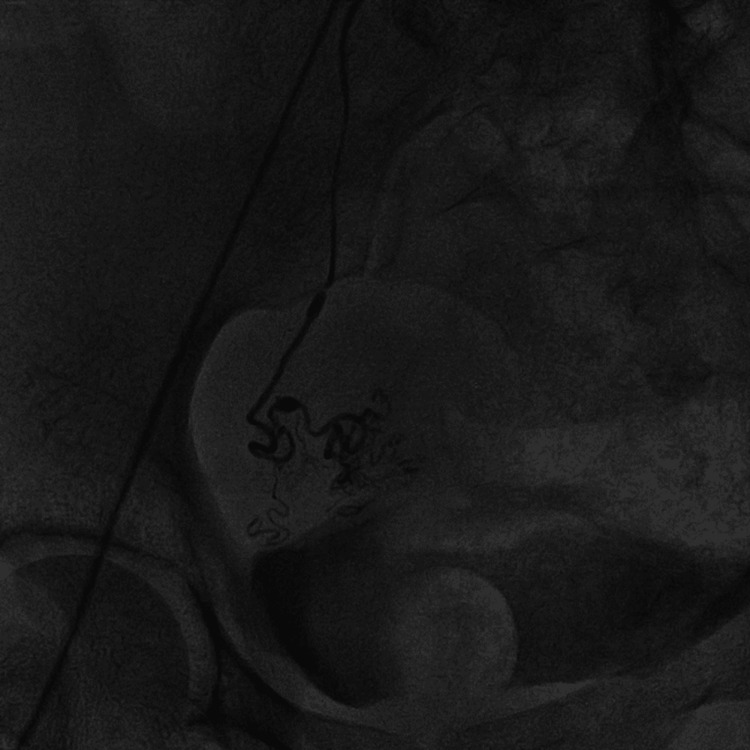
Embolization of the right uterine artery branch done using polyvinyl alcohol

**Figure 3:**
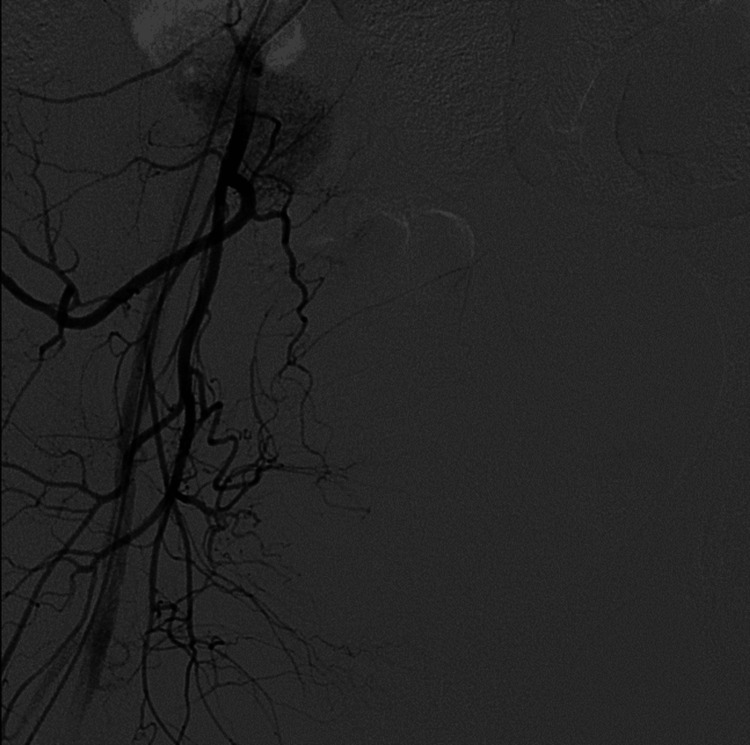
No spillage of dye post embolization of right uterine artery branch

CT angiography of the right internal iliac artery was done which revealed a spilling of dye from the branches of the right uterine artery and a small aneurysm found in one of the branches (Figure 1). On the basis of angiography findings, right-sided uterine artery embolization under local anaesthesia was done using polyvinyl alcohol foam (Figure 2). Post embolization, there was no spillage of dye through the uterine artery branches (Figure 3).

Postoperatively, the patient was vitally stable and her vitals one hour following embolization were: BP 110/72 mmHg with a pulse rate of 98 bpm and SpO2 of 100% on room air. On per vaginal examination, she had no evidence of bleeding. The postoperative course was uneventful and the patient was discharged on day 4 of UAE without any complications. On discharge, her anaemia and leukocytosis were resolved.

## Discussion

Postoperative haemorrhage occurring after any surgery is one of the most common complications causing morbidity and overall mortality in hospitals. Moreover, it also increases the duration of hospital stay and the overall cost of healthcare.

Abdominal hysterectomy is one of the most commonly done procedures in obstetric surgery. However, all surgical procedures are associated with related risks and complications. Abdominal hysterectomy may be associated with various perioperative and postoperative adverse outcomes like haemorrhage, urinary incontinence [6], decreased sexual function [7], early onset of menopause, keloid formation, etc.

The common causes of postoperative bleeding include improperly ligated vessels, dislodgement of clots, hypertension, improper surgical technique, pressure necrosis, subacute infection and coagulation disorders [8]. Surgical re-exploration was traditionally used to control secondary postoperative haemorrhage. However, it was often associated with surgical complications like infection, bleeding, ureter injury and the pertinent surgical and anaesthetic risks associated with emergency operations. Haemodynamically unstable patients had relatively more chances of anaesthetic complications. During surgical exploration, bleeding vessels were identified and ligated. Since the pelvis is supplied by extensive anastomosis of blood vessels, the chances of successful control of bleeding were limited if the bleeding sites were extensive or unidentifiable or if there is anatomical inaccessibility of bleeding vessels. Inflammatory reactions including pelvic adhesions are also responsible for the failure of surgical explorations.

Transcatheter arterial embolization used in our patient to treat secondary postoperative bleeding is emerging as an effective percutaneous technique to control peritoneal and genital bleed in various obstetric and gynaecological procedures with minimal surgical trauma and morbidity. Pelvic digital subtraction angiography has very high sensitivity in detecting bleeding sites. Angiography gives the advantage of therapeutic embolization along with precise identification and localisation of bleeding vessels. [9]

However, as with every surgical procedure, transarterial embolization may be associated with complications like pelvic pain, low-grade fever, nausea, vomiting, loss of appetite, post-embolization syndrome, etc. Rare complications include iliac thrombosis, ischemic neuropathy, bladder gangrene, uterine necrosis, leg ischemia, and buttock necrosis [10], and the operating surgeon needs to be aware and cautious of these possible complications.

## Conclusions

To conclude, angiography-assisted transcatheter arterial embolization is a safe and minimally invasive option to manage patients with postoperative haemorrhage as observed in our case of postoperative secondary haemorrhage following hysterectomy.
